# Factors associated with false positive results in serological testing for syphilis using EIA among children

**DOI:** 10.3389/fped.2025.1671397

**Published:** 2025-11-11

**Authors:** Jiaqi Liu, Qianqian Chen, Guangchao Zhao, Xuzhou Fan, Wei Wang

**Affiliations:** 1 Department of Blood Transfusion, Jinling Hospital, Affiliated Hospital of Medical School, Nanjing University, Nanjing, Jiangsu, China; 2 Department of Blood Transfusion, The Affiliated Suzhou Hospital of Nanjing Medical University, Suzhou Municipal Hospital, Suzhou, Jiangsu, China; 3 Department of Laboratory, Suzhou Xiangcheng Center for Disease Control and Prevention, Suzhou, Jiangsu, China

**Keywords:** adenoidal hypertrophy (AH), syphilis infection, false-positive result, coagulationprofile, systemic inflammatory indicators

## Abstract

**Objectives:**

The issue of biological false positives in syphilis diagnosis is gaining attention. However, limited focus exists on false positives in syphilis tests among younger populations. This study investigates the epidemiological characteristics and influencing factors of false positive serological test results for syphilis in children.

**Methods:**

A retrospective study was conducted on the serological test results, demographic, clinical, and laboratory characteristics of children in Jinling Hospital from 2017 to 2022.

**Results:**

This study included 18 cases of false positive syphilis results. Children aged 5–9 years had a higher proportion of false positives compared to those with negative results (*p* < 0.001). The prevalence of adenoid hypertrophy (AH) in children with false positives was 38.89%, significantly higher than in those with negative results (*p* < 0.0001). The results of syphilis antibodies detection by enzyme-linked immunosorbent assay (EIA) in false positive cases, negative cases, and true positive cases were significantly different (*p* < 0.0001). Children with false-positive syphilis results had significantly higher systemic immune-inflammation index (SII), fibrin degradation products (FDP), and platelet (PLT) levels than those with negative results (*p* < 0.05). Compared to true-positive cases, false-positive cases showed lower systemic inflammation response index (SIRI) and neutrophil-to-lymphocyte ratio (NLR) but higher lymphocyte-to-monocyte ratio (LMR), antithrombin III (AT-III), and PLT levels (*p* < 0.05). Furthermore, among children with false-positive serological test results for syphilis, certain coagulation parameters, such as FDP, D-dimer (DD), AT-III, and PLT, were found to be elevated (*p* < 0.05). Univariate logistic regression analysis revealed that age (OR = 0.852, 95% CI: 0.766, 0.948), AH (OR = 20.10, 95% CI: 5.361, 79.53), APTT (OR = 0.804, 95% CI: 0.658, 0.977), FDP (OR = 1.722, 95% CI: 1.234, 2.416), AT-III (OR = 1.071, 95%CI: 1.030, 1.121), and PLT (OR = 1.008, 95%CI: 1.003, 1.013) were risk factors associated with the occurrence of a false positive reaction in syphilis serology (*p* < 0.05).

**Conclusions:**

In the assessment of false-positive syphilis test results, age and inflammatory marker data exhibit reference value. AH and partial coagulation function indices are risk factors for false positive syphilis serology results in children. Therefore, it is crucial for clinical and laboratory doctors to pay close attention to positive results for syphilis in such cases.

## 1 Introduction

Syphilis is a chronic systemic sexually transmitted infection caused by *Treponema pallidum* (TP), characterized by a highly infectious and intricate clinical course, which can be transmitted through sexual contact, blood transfusion and maternal-neonatal transmission (1). According to estimates by the World Health Organization (WHO), there are over 12 million new cases of syphilis globally each year, with approximately 8 million cases reported among adults in 2022 (2). The global syphilis epidemic remains a significant public health concern. From 2004 to 2019, a total of 5,527,399 syphilis cases were reported in China, with an annual average incidence rate of 25.7063 cases per 100,000 population. The data indicate a general upward trend over the study period (3). Syphilis has emerged as a significant global public health concern. Additionally, the increasing incidence of syphilis among women has led to a higher prevalence of congenital syphilis, thereby elevating the likelihood of children acquiring the infection (4).

**Figure 1:**
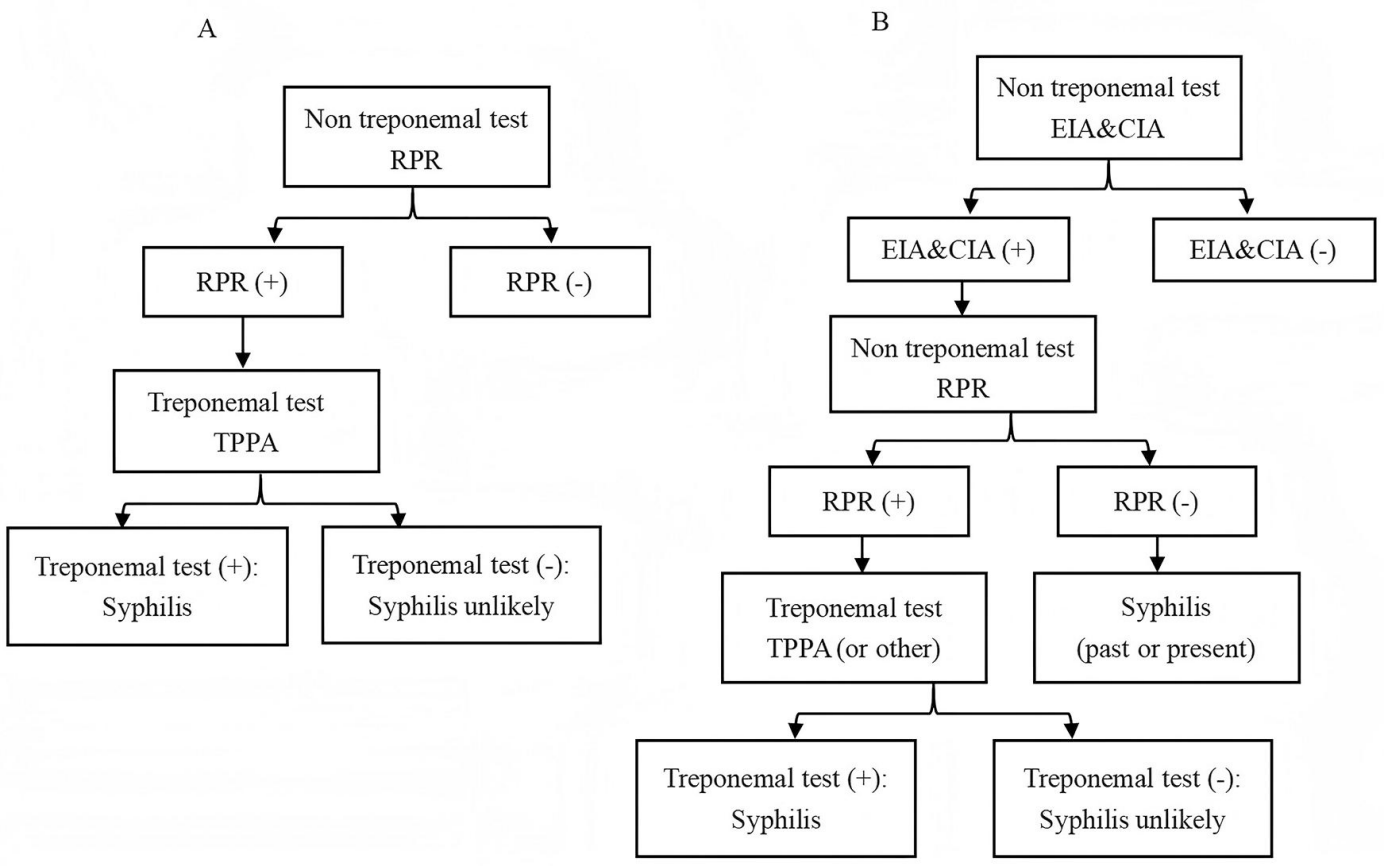
Testing algorithms for syphilis diagnosis. **(A)** Traditional. **(B)** Reverse. EIA, enzyme-linked immunosorbent assay; CIA, chemiluminescence immunoassays; RPR, rapid plasma reagin; TPPA, treponema pallidum particle agglutination.

Currently, the laboratory diagnosis of syphilis primarily relies on serological tests, which can be categorized into treponemal antibody tests and non-treponemal antibody tests (5). The treponemal antibody tests, such as enzyme-linked immunosorbent assay (EIA) and chemiluminescence immunoassay (CIA), remain widely utilized in laboratory settings due to their exceptional sensitivity and specificity for detecting treponemal antibodies. These tests are extensively employed as the primary screening method for blood donors (6). Traditionally, serological testing for syphilis begins with the use of non treponemal tests such as the toluidine red unheated serum test (TRUST) and rapid plasma reagin (RPR) (7). The specific treponemal test, such as treponema pallidum particle agglutination (TPPA), was then used for confirmation, as shown in Figure 1A. Recently, to enhance the accuracy of syphilis screening results, a “reverse algorithm” has been proposed. This approach incorporates an initial specific treponemal antibody test, such as EIA or CIA, prior to the conventional screening strategy (8). The detailed procedure is illustrated in Figure 1B.

Despite advancements in syphilis screening strategies, serological tests for syphilis continue to exhibit certain limitations, including a limited correlation with disease activity (9). Moreover, the issue of biological false positives in syphilis detection has also garnered increased attention (10). The elderly population may experience false positive results in syphilis serological tests due to factors such as disease status, administration of drugs, and viral infection (11–13). However, there is relatively less concern regarding the occurrence of false positive reactions in syphilis serology among children, who are a vulnerable group. The present study aimed to investigate the occurrence of false-positive results in TP-EIA tests among children before blood transfusion. Additionally, it sought to identify the risk factors associated with these cases and further analyze potential causes. The ultimate goal was to ensure accurate clinical diagnosis and minimize disputes by providing valuable references.

## 2 Materials and methods

### 2.1 General information

**Figure 2:**
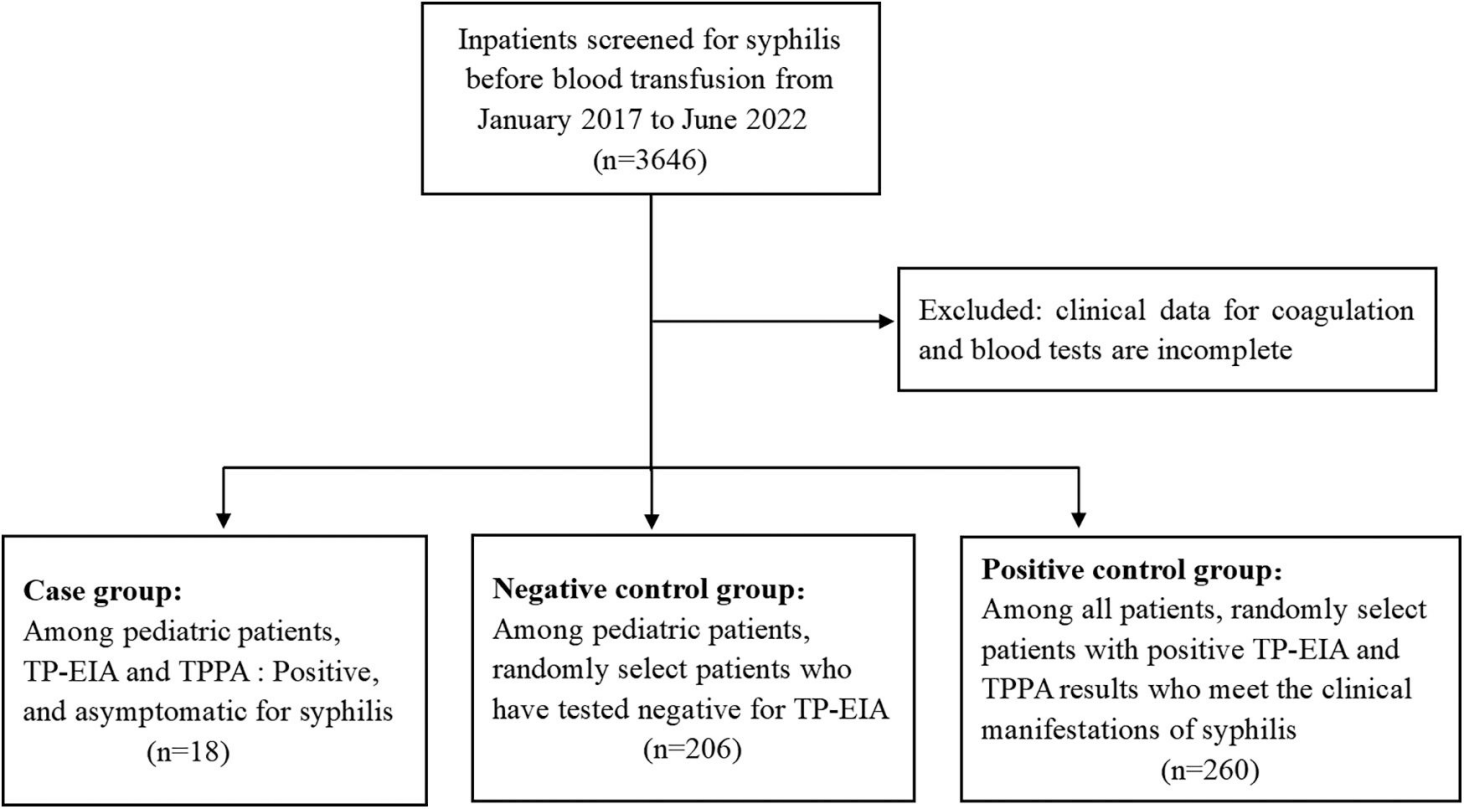
Flowchart of the study cohort.

Children who exhibited false-positive syphilis serological test results were identified through the reverse algorithm of syphilis testing among those planned to receive blood transfusions, surgery, or other treatments at Jinling Hospital between January 2017 and June 2022. The physical examination findings were within normal limits in all cases, including normal skin color, absence of jaundice, and normal skin temperature and moisture levels. None of the patients reported a history of hepatitis, tuberculosis, malaria, asthma, surgery, trauma or blood transfusion. Additionally, there was no clear evidence of drug allergies. The control group in this study comprised 206 individuals who tested negative for syphilis serologically and were randomly selected from the children during the same time period. The demographic data, clinical data, and coagulation function results of the above two groups were collected, including prothrombin time (PT), activated partial thromboplastin time (APTT), plasma thrombin time (TT), fibrinogen (FIB), fibrin degradation product (FDP), D-dimer (DD), antithrombin III (AT-III) and platelet (PLT). Additionally, 260 adult patients who were clinically diagnosed with syphilis during the same period were randomly selected. Their TP-EIA results and clinical examination data were collected for inclusion in the positive control group. Figure 2 presents the flowchart of the study cohort. All strategies conducted in this study involving human participants were in accordance with the ethical standards of the institutional and/or national research committee and with the 1964 Helsinki declaration and its later modifcations or comparable ethical standards. This study was approved by the Ethics Committee of Jinling Hospital.

### 2.2 Systemic inflammatory indicators

The levels of systemic inflammatory indicators, including the white blood cell (WBC) count, systemic inflammation response index (SIRI), lymphocyte-to-monocyte ratio (LMR), systemic immune-inflammation index (SII), and neutrophil-to-lymphocyte ratio (NLR), were assessed based on the respective cell counts obtained from routine blood tests performed concurrently with syphilis screening. The calculation was carried out in accordance with the formula presented below: SIRI=MONO×NEUT/LYMPH,LMR=LYMPH/ MONO,SII=PLT×NEUT/LYMPH,NLR=NEUT/LYMPH.

### 2.3 Instruments and reagents

Serum specimens were assessed for anti-TP by EIA on the FAME24/20 (Hamilton, Switzerland). EIA was performed using products from InTec Inc Company and approved by the State Food and Drug Administration of China. TPPA (Serodia, Tokyo, Japan) experiments were conducted in the patients with EIA positive for further verification. The coagulation function was detected by automatic coagulation analyzer (Sysmex CS5100; Wakinohama-Kaigandori, Japan). All experimental steps were carried out according to the manufacturers' information.

### 2.4 Serologic assays

All the results of EIA for syphilis antibodies in all specimens were expressed as signal-to-cutoff (S/CO) ratio. When S/CO < 1, it indicated a nonreactive result and vice versa. Similarly, the positive, negative, and blank controls were signed. The sample was tested using double-well potential, and the results all reached S/CO ≥ 1 and were judged as positive. The TPPA results can be interpreted as negative, weakly positive, or positive, based on the manufacturer's provided interpretation note. Negative results are when no agglutination is visible, positive results are when agglutination is obvious, and weak positive results are when agglutination has occurred but not completely. All reagents were from quality batches and were used within the validity period.

### 2.5 Interpretation of assay results

In this study, the determination of false positive results in syphilis serological testing was based on TPPA as a confirmatory assay, and was further evaluated in conjunction with clinical manifestations to reach a final assessment. Specifically, according to the results of the EIA and TPPA assays, false-positive reactions were defined as samples that tested positive by EIA but negative by TPPA, with clinical manifestations not consistent with syphilis. True-positive reactions were defined as cases where patients exhibited positive results in both EIA and TPPA. A negative response was characterized by negative results in EIA.

### 2.6 Statistical analysis

All statistical analyses were performed using the GraphPad Prism software 9.4.1 (GraphPad Software, San Diego, CA, USA). Continuous variables with normal distribution were reported as mean ± standard deviation and compared using *t*-tests; non-normally distributed data were presented as median and interquartile range (IQR) and analyzed with the Mann–Whitney *U* test. Categorical variables were expressed as percentages and evaluated using the chi-square test for statistical inference. Univariate and multivariate logistic regression were used to analyze the risk factors for false positive results of syphilis serology. Candidate variables with a *p* value less than 0.2 in the univariate analysis were included in the multivariable model. Variables with a *p* value of less than 0.05 were considered statistically significant.

## 3 Results

### 3.1 Demographic characteristics of the study participants

A total of 224 cases were included in this investigation, among which 18 exhibited false-positive syphilis antibody test results, with a median age of 9.00 (5.75–10.25) years old. The diagnoses comprised 4 cases of Henoch-Schonlein purpura (HSP), 7 cases of adenoid hypertrophy (AH), 3 cases of renal disease, 2 cases of orthopedic disease, and 2 cases of neurosurgical disease. Among the remaining 206 children who tested negative for syphilis antibodies, their median age was 13.00 (9.00–16.00) years old. As a result, the mean age of children exhibiting false-positive syphilis test results was significantly lower compared to that of patients with negative test results (*p* < 0.001). The false positive group consisted of 8 males (44.44%) and 10 females (55.56%), while the negative group comprised of 79 males (38.35%) and 127 females (61.56%). This study examined differences in age distribution between children with false positive results and those with negative results. The findings indicated that in the 5–9 years old and 9–13 years old groups, the proportions of false positive cases were 33.33% and 38.89%, respectively, which were significantly higher than the corresponding proportions of 16.02% and 23.30% observed in the negative group. The difference in the 5–9 years old group was statistically significant (*p* < 0.05). In contrast, within the 13–18 years old group, the proportion of false positive cases was 16.67%, substantially lower than the 53.88% reported for the negative group (*p* < 0.001). Furthermore, in terms of comparisons among different age groups, the number of false positive cases among children aged 5–9, 9–13, and 13–18 years old was 6 (15.38%), 7 (12.72%), and 3 (2.63%) respectively, which also indicates that the age group of 5–13 years old has a higher false positive rate. Among children with false-positive syphilis test results, the proportions of AH and HSP are 38.89% and 22.22% respectively, which are significantly higher than those among children with negative syphilis test results (*p* < 0.05), as shown in Table 1.

**Table 1:** The demographic characteristics distribution for the study cohort.

Categories	Patients with false-positive syphilis serology results	Patients with negative syphilis serology results	*χ* ^2^	*p*
Number of participants, (*n* = 224)	18	206		
Age [year; median (IQR)]	9.00 (5.75–10.25)	13.00 (9.00–16.00)	—	<0.01
Age (year), *n* (%)				
0–5 (*n* = 16)	2 (11.11)	14 (6.80)	0.465	0.495
5–9 (*n* = 39)	6 (33.33)	33 (16.02)	3.451	<0.05
9–13 (*n* = 55)	7 (38.89)	48 (23.30)	2.171	0.141
13–18 (*n* = 114)	3 (16.67)	111 (53.88)	9.174	<0.001
Gender, *n* (%)				
Male (*n* = 87)	8 (44.44)	79 (38.35)	0.259	0.611
Female (*n* = 137)	10 (55.56)	127 (61.65)		
Types of disease, *n* (%)				
Adenoid hypertrophy (*n* = 12)	7 (38.89)	5 (2.43)	43.41	<0.0001
Henoch-Schonlein purpura (*n* = 20)	4 (22.22)	16 (7.76)	4.254	<0.05
Renal disease (*n* = 120)	3 (16.67)	117 (56.80)	10.72	<0.01
Neurosurgical disease (*n* = 22)	2 (11.11)	20 (9.71)	0.048	0.827
Orthopedic disease (*n* = 15)	2 (11.11)	13 (6.31)	0.611	0.435
Others (*n* = 35)	0 (0)	35 (16.99)	3.709	0.054

### 3.2 Results of EIA for syphilis antibodies

**Figure 3:**
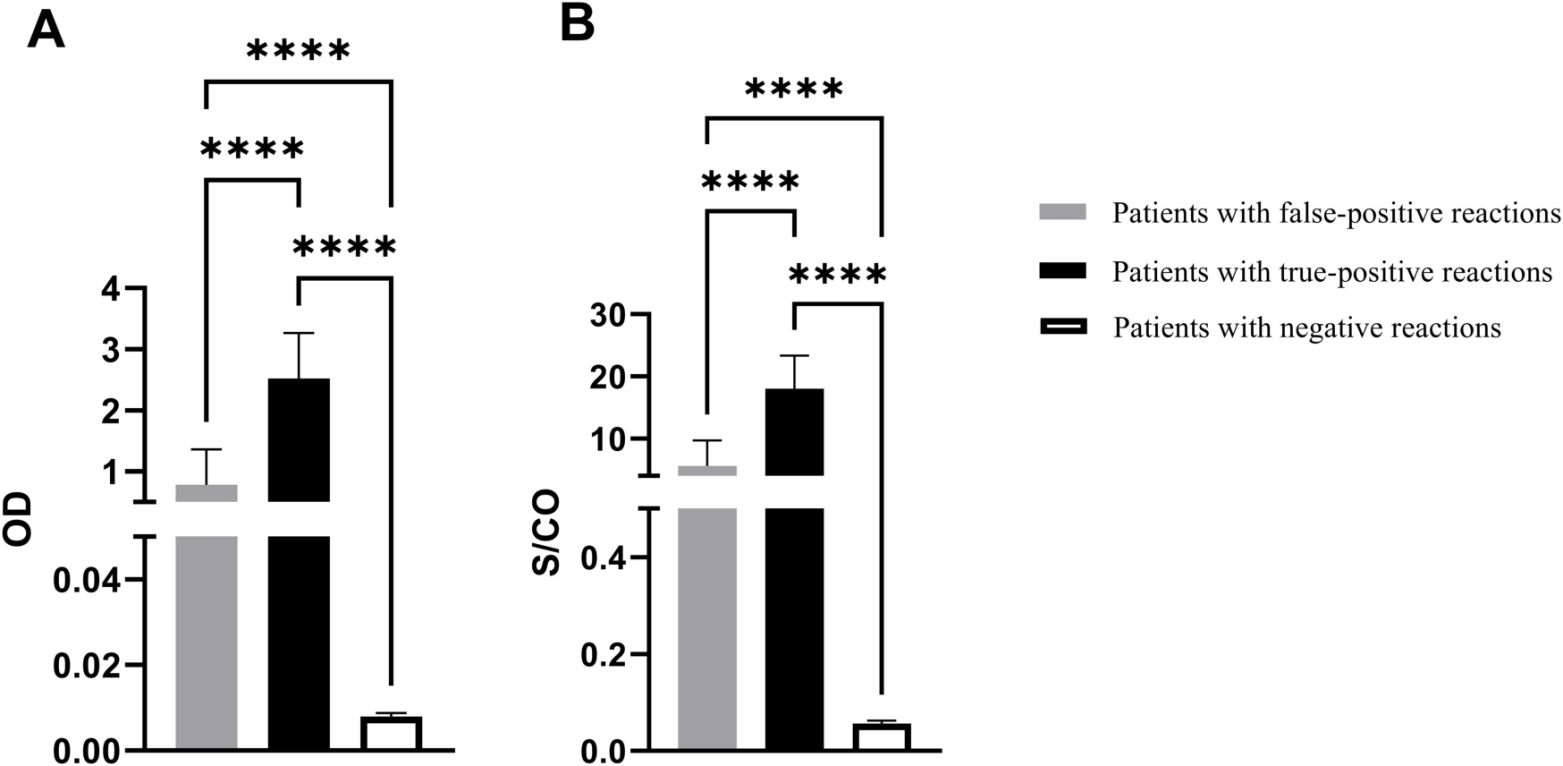
The EIA results for syphilis antibodies. **(A)** OD value of syphilis antibody test. **(B)** S/CO ratio. OD: optical density; S/CO: signal-to-cutoff; ^****^p < 0.0001.

The optical density (OD) values and S/CO ratios among children exhibiting false-positive syphilis results were 0.782 ± 0.578 and 5.59 ± 4.13, respectively, whereas the corresponding values for syphilis negative children were 0.008 ± 0.001 and 0.057 ± 0.005. For patients with true-positive syphilis, OD values and S/CO ratios were recorded at 2.664 ± 0.660 and 19.03 ± 4.71, respectively. There were significant differences among the three groups (*p* < 0.0001), as shown in Figure 3.

### 3.3 Systemic inflammatory indicators and coagulation status in patients

**Figure 4:**
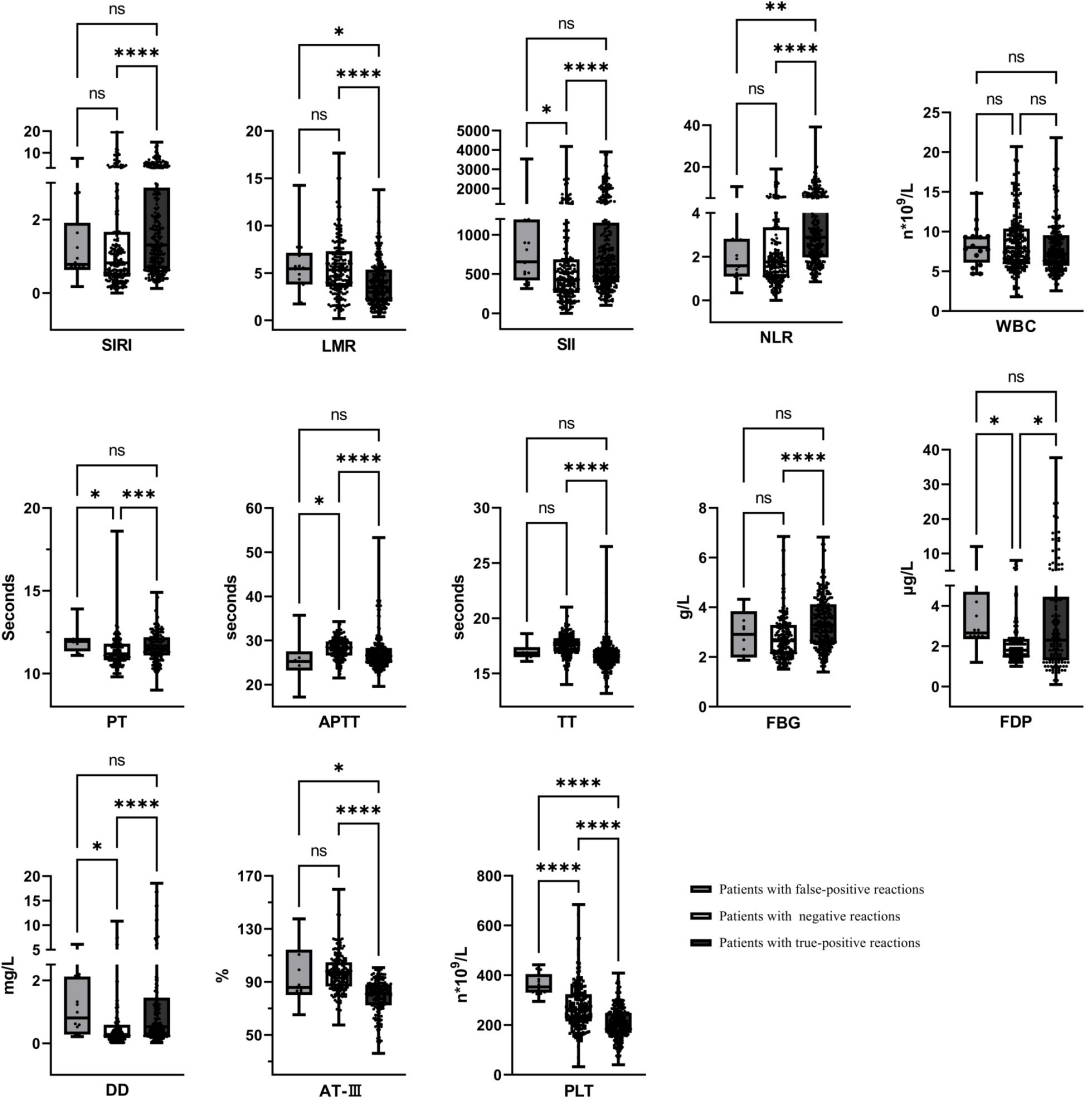
Systemic inflammatory indicators and coagulation status in patients. ^*^p < 0.05, ^**^p < 0.01, ^***^p < 0.001, ^****^p < 0.0001, ^ns^*p* > 0.05.

The systemic inflammatory indicators and coagulation function changes were retrospectively analyzed in 18 cases with false serological positivity, 206 cases with negative syphilis test results, and 260 cases with true-positive syphilis. Children with false-positive syphilis test results exhibited significantly higher levels of SII, FDP, and PLT counts compared to those with negative test results (*p* < 0.05). When compared to children with true-positive syphilis test results, those with false-positive results demonstrated lower levels of SIRI and NLR, but markedly elevated levels of LMR, AT-III, and PLT counts (*p* < 0.05), as shown in Figure 4.

### 3.4 Analysis of risk factors for serological false positives of syphilis

A syphilis serological false-positive result was designated as the dependent variable, while age, gender, AH, HSP, systemic inflammatory markers, and coagulation function parameters were selected as independent variables. Univariate and multivariate logistic regression analysis were used to conduct correlation analysis. Univariate logistic regression analysis revealed that age (OR = 0.852, 95% CI: 0.766, 0.948), AH (OR = 20.10, 95% CI: 5.361, 79.53), APTT (OR = 0.804, 95% CI: 0.658, 0.977), FDP (OR = 1.722, 95% CI: 1.234, 2.416), AT-III (OR = 1.071, 95%CI: 1.030, 1.121), and PLT (OR = 1.008, 95%CI: 1.003, 1.013) were risk factors associated with the occurrence of a false positive reaction in syphilis serology (*p* < 0.05). Age, AH, HSP, PT, APTT, FDP, AT-III, and PLT were included in the multivariate regression analysis. The results indicated that APTT (OR = 0.753, 95% CI: 0.570, 0.996) was a significant independent predictor (*p* < 0.05). There were no statistically significant differences observed in the remaining indicators (*p* > 0.05), as shown in Table 2.

**Table 2:** Logistic regression analysis of the factors affecting the serological false positive of syphilis.

Categories	Univariate analysis			Multivariate analysis		
	OR	95% CI	*p*	OR	95% CI	*p*
Age (years)	0.852	0.766, 0.948	<0.01	0.737	0.541, 1.004	0.053
Gender						
Male	1	—	—			
Female	1.286	0.473, 3.397	0.612			
Types of disease						
AH (vs. non-AH)	20.10	5.361, 79.53	<0.01	3.765	0.105, 134.6	0.468
HSP (vs. non-HSP)	3.392	0.884, 10.82	0.050	2.818	0.181, 43.85	0.459
Laboratory data						
SIRI	0.969	0.756, 1.057	0.704			
LMR	1.015	0.874, 1.159	0.830			
SII	1.000	0.999, 1.000	0.884			
NLR	0.983	0.807, 1.030	0.769			
WBC (10^9^/L)	1.054	0.921, 1.194	0.421			
PT (s)	1.864	0.953, 3.521	0.057	1.813	0.486, 6.760	0.376
APTT (s)	0.804	0.658, 0.977	<0.05	0.753	0.570, 0.996	<0.05
TT (s)	0.710	0.393, 1.283	0.255			
FIB (g/L)	0.791	0.367, 1.418	0.491			
FDP (μg/L)	1.722	1.234, 2.416	<0.01	1.261	0.755, 2.435	0.119
DD (mg/L)	0.969	0.493, 1.152	0.856			
AT-III (%)	1.071	1.030, 1.121	<0.01	1.040	0.980, 1.104	0.198
PLT (10^9^/L)	1.008	1.003, 1.013	<0.01	1.011	0.996, 1.026	0.154

## 4 Discussion

In recent years, the occurrence of false positive results in syphilis serological testing, particularly in non-treponemal assays, has garnered growing attention in clinical practice. According to a Japanese investigative report on biological false positives (BFPs) in syphilis testing, among 94,462 individuals examined, a total of 588 cases (0.62%) exhibited BFPs, with the majority concentrated in the approximately 60-year-old age group (10). It has been reported that syphilis BFPs are associated with certain characteristics of the patient, such as gender (female), age (elderly), intravenous drug use, pregnancy, and the presence of other autoimmune diseases, including systemic lupus erythematosus (SLE), cancer, malaria, other spirochetal infections, human immunodeficiency virus (HIV) infection, and hepatitis C infection, among others (14–16). However, research on false positive outcomes associated with syphilis-specific antibody detection methods, such as EIA and CIA, remains relatively limited. For example, a study conducted in Brazil reported that among blood donors initially screened positive for syphilis using CIA, the false positive rate reached 18.33% following confirmatory testing (17). Another study indicated that the specificity of EIA is approximately 98%, implying that a non-negligible proportion of false positive results still occur (18). Furthermore, certain studies have explored approaches to reduce false positive reactions in Treponema pallidum-specific antibody detection via urea-mediated dissociation techniques in EIA (19). Serological false positives for syphilis can pose challenges in monitoring and diagnosing syphilis. At present, the elderly population has been reported as a risk factor for false positive syphilis, while the serological false positive of syphilis in minors has received less attention. Therefore, this study focused on the serological false positives of syphilis in children, aiming to provide reference for improving the accuracy of screening and diagnosis of syphilis in children.

In this study, AH accounted for a higher proportion of cases in children with false positive serologic syphilis, suggesting that AH may be a risk factor for false positive syphilis. The adenoids, also known as the pharyngeal tonsils, are part of the pharyngeal lymphatic ring located in the back wall of the nasopharynx, and are also one of the organs of the body's immune system (20). Different stages of lymphocytes are produced in adenoids, which play an important role in humoral and cellular immunity (21). AH is associated with pathogen infection and local immune dysfunction, ultimately characterized by abnormal quantity and function of multiple lymphocyte subsets within the adenoids, leading to the continuous release of a large number of inflammatory mediators and substances related to coagulation dysfunction (22). The EIA immunoassay is based on the specific binding between antigens and antibodies and is primarily used in clinical settings to detect antigens, antibodies, or immune cells in biological samples. However, various interfering factors in clinical samples can compromise the accuracy of EIA results (23). For instance, rheumatoid factor (RF) can bind to both the solid-phase antibody and the enzyme-labeled reagent, triggering a colorimetric reaction that leads to false-positive outcomes (24). Additionally, substances with structurally similar epitopes or highly homologous antigenic determinants may cause cross-reactivity. For example, the *β* subunit of human chorionic gonadotropin (HCG) shares high homology with luteinizing hormone (LH), serine protease, and transforming growth factor beta (TGF-*β*), potentially resulting in falsely elevated HCG measurements (25). When patients are receiving anticoagulant therapy and exhibit impaired coagulation function, elevated fibrin levels may lead to a falsely increased measurement of thyroid hormone levels (26). Our study suggests that false-positive results in syphilis EIA tests among children may be attributable to endogenous interfering factors present in their serum. Therefore, clinicians and laboratory personnel should pay attention to syphilis positive results in children with AH.

HSP is one of the most common immune complex-mediated vasculitis in children, with a prevalence of about 13–20/100,000 children (27). The pathogenesis of HSP is still unclear. It is often thought to be systemic small vasculitis caused by bacterial, viral, and other allergens. A large amount of IgA immune complex deposition can be detected in the walls of small blood vessels, leading to aseptic vasculitis and even inducing necrotizing arteriitis (28). It has been reported that the levels of factor VIII and homocysteine in patients with HSP are significantly increased, resulting in a hypercoagulable state and an increased risk of thrombosis (29). In this study, the levels of FDP and PLT in false-positive HSP children were significantly higher than those in negative children. Given that a large number of specific FDP exist in false positive children, and TP-EIA is more susceptible to interference from fibrin and other substances, resulting in cross-reaction (14). Therefore, we speculate that the abnormal increase of fibrin and its degradation products may be related to the positive reaction of TP-EIA. In addition, this study found that FDP, AT-Ⅲ, and PLT levels were significantly associated with false-positive results in syphilis serology, suggesting that these factors may contribute to an increased risk of false-positive outcomes.

In the cases of children with false-positive syphilis tests identified in this study, most were diagnosed with immune-related diseases, and inflammatory reactions commonly occur during the progression of such conditions (30). Therefore, we focused on the changes in systemic inflammatory markers in these children. Previous studies have demonstrated that systemic inflammatory indices, such as the SIRI, the LMR, the SII, and the NLR, serve as effective biomarkers for assessing the body's inflammatory status and have been widely applied in the diagnosis and evaluation of various diseases (31). Specifically, elevated levels of SIRI, SII, and NLR, along with a reduced LMR, are generally considered indicators of an enhanced inflammatory response (32). According to relevant reports, the average SII value in infants with febrile urinary tract infection (UTI) complicated by severe bacterial infection was 795.76, which was significantly higher than the value of 318.24 observed in the group without bacterial infection. These findings suggest that SII levels may serve as a potential biomarker for predicting the presence of bacteremia in children with UTI (33). In a study involving children with pneumonia, it was observed that the PLR, SII, and SIRI values in children diagnosed with necrotizing pneumonia (NP) were significantly elevated compared to those in children with community-acquired pneumonia (CAP) and parapneumonic effusion (34). Therefore, these systemic inflammatory markers may hold significant potential for application in the diagnosis and prediction of infectious or inflammatory diseases in children. Accordingly, we incorporated these indices to explore potential risk factors associated with false-positive syphilis tests in children. Our findings revealed that the SII level in children with false-positive results was significantly higher compared to those with negative tests, whereas SIRI and NLR levels were markedly lower than those observed in patients with confirmed syphilis infections. These results suggest that the inflammatory response in children with false-positive syphilis tests is stronger than in those with negative tests, but weaker than in those with true syphilis infections. These observations provide valuable insights for evaluating the actual clinical status of children with false-positive syphilis test results.

Our study has certain limitations. The retrospective analysis method used in this study may be subject to selection bias. Although the present study has identified an association between coagulation function parameters, inflammatory indicators, and false-positive syphilis tests, it cannot establish causality. A prospective study design, along with long-term follow-up data, would be necessary to establish causal relationships. This study has certain limitations regarding the selection method of research participants. The false positive cases observed in the article may be associated with potential immune system disorders, and this finding warrants further validation in more children. In addition, due to the limited availability of specimen resources, this study used adult positive samples for comparative analysis instead of pediatric syphilis cases. Given the differences between children and adult populations in immune system development, physiological metabolism, and disease pathogenesis, future validation studies should be conducted using positive samples obtained from children. In this study, the number of children in the syphilis false positive group is relatively small. Compared with other groups, this limitation may introduce potential biases into the statistical analysis. Future research should prioritize expanding the sample size and conducting multi-center, large-sample studies for validation, thereby enhancing the representativeness and reliability of the research findings.

## 5 Conclusions

In summary, syphilis serological testing has certain limitations, and the results may be biased in people with different diseases and ages. In recent years, there have been increasing reports of false positive syphilis tests in elderly patients. However, the results of syphilis test in young people are less concerned, and children are a special group with more social sensitivity, so inspectors should pay more attention when reporting results. In cases of AH, HSP, or other immune-related diseases reported in this study, when seropositive results for syphilis are observed, a comprehensive analysis should be conducted in conjunction with systemic inflammatory indicators and coagulation function parameters in children. When conditions are available, it is recommended to use a more specific syphilis immunoblotting method for further confirmation, and carefully send experimental results based on clinical manifestations.

## Data Availability

The original contributions presented in the study are included in the article/Supplementary Material, further inquiries can be directed to the corresponding author.
